# Retention and attrition of community health volunteers in rural Malawi; a mixed-methods study

**DOI:** 10.1186/s12889-026-28321-0

**Published:** 2026-07-01

**Authors:** Blessings N. Kaunda-Khangamwa, Harrison Msuku, Cecilia T. Kamiza, Alfred A. Chimala, Christopher C. Stanley, Jobiba Chinkhumba, Victor Mwapasa, Don P. Mathanga

**Affiliations:** 1 School of Global and Public Health, Department of Bioethics and Behavioural Social Sciences, Kamuzu University of Health Sciences, P/Bag 360, Mahatma Gandhi, Blantyre, Malawi; 2 MAC-Communicable Diseases Action Centre (MAC-CDAC), Kamuzu University of Health Sciences, P/Bag 360, Mahatma Gandhi, Blantyre, Malawi; 3 School of Public Health, University of the Witwatersrand, 27 St Andrews Road, Parktown, Johannesburg, 2193 South Africa

**Keywords:** Attrition, Community Health Volunteers (CHVs), Malaria Vaccine Implementation Program (MVIP), Malawi, Retention, Volunteering

## Abstract

**Background:**

Community health volunteers (CHVs) are an important resource for supporting health service delivery, surveillance, and social programmes. However, retention and attrition of CHVs remain a big challenge. This study explored factors affecting the retention and attrition of CHVs working as village reporters (VRs) responsible for community-based death notification in the Malaria Vaccine Implementation Program (MVIP) in Malawi.

**Methods:**

This mixed-methods exploratory study, which intersected with the case studies, was conducted from November 2022 to March 2023 in nine rural districts in southern and central Malawi. Purposive sampling was used to select 64 study participants for qualitative interviews. Using case studies, we conducted six in-depth interviews (IDIs) with CHVs who had dropped out, were reachable, and agreed to be interviewed—many were dispersed, hesitant to attend meetings, or unreachable for focus group discussions (FGDs). We held five FGDs (*n* = 50) with CHVs who remained in the MVIP for shared norms and experiences and eight key informant interviews (KIIs) with health workers, opinion leaders, and program staff to provide insights into health workers motivation, supervisory and program perspectives. Thematic analysis and the social capital framework (roles, relationships, and empowerment) were used to analyse and interpret the qualitative data. The qualitative study was complemented by a cross-sectional survey involving 696 randomly selected participants from a pool of 2,861 CVHs to demonstrate the trends of retention over time. Descriptive statistics were computed from quantitative survey data, with retention as a primary outcome (defined as whether a CHVs was willing to stay in the program).

**Results:**

At the start of the program in 2019, a total of 2,861 CHVs were recruited by March 2023; only 295 (10.3%) had dropped out. Among 696 CHVs surveyed, the most commonly reported factors associated with retention were incentives (643; 92%), participation in exchange visits (377; 54%), and managing a small geographical area (275; 40%). Qualitative data from FGDs and KIIs corroborated these findings and identified compassion for serving others, financial and non‑financial incentives, and flexibility to work across multiple programmes as key motivators for continued participation. Factors associated with attrition included experiences of ridicule or disrespect, lack of opportunities for personal development, and limited career progression. IDIs with CHVs who left the programme provided in‑depth accounts of these individual‑level drivers, which helped explain patterns observed in the survey.

**Conclusion:**

Engagement of CHVs in community-based programs can be promoted by offering opportunities to serve others, incentives, and flexibility to work on multiple programmes. However, it is also important to address ridicule—making fun or rude comments and limited personal and career development, which act as barriers to the continued engagement of lay health workers.

**Supplementary Information:**

The online version contains supplementary material available at https://doi.org/10.1186/s12889-026-28321-0.

## Introduction

Low-income countries in Africa face severe shortages of health personnel that critically affect healthcare systems [1–3]. Nevertheless, community health volunteers (CHVs), with appropriate task shifting, are effective in strengthening health systems by improving access to health services, reducing demand for trained health workers, and reducing morbidity and mortality, especially in rural hard-to-reach areas [4–6].

Despite the wide participation of CHV in the delivery of health and social programs in Africa, many do not receive any remuneration and some programmes are underfunded. In some resource-poor settings, attrition rates are high, with first‑year attrition between 3.2% and 5%, and cumulative attrition over 2–5 years reaching up to 77.0%, leading to frequent service delivery disruptions [7–9]. Research indicates that, beyond incentives—which cover various tokens, gifts, awards, commendations, and payments aimed at motivating volunteers, including factors that discourage volunteers from working for the community’s well-being include: (1) limited opportunities for integration into existing structures or programs, (2) insufficient information about interventions and implementation processes, and (3) under-reporting of the CHV selection processes, all of which are linked to higher attrition rates [10–12].

In Malawi, CHVs are defined as “individuals who willingly offer their time, skills, and knowledge to work with the communities to improve the health status of the communities where they reside without expecting financial remuneration” [5, 13]. CHVs are volunteers involved in community health activities. VRs are a subset, chosen from Village Health Committees to support the MVIP. With health worker shortages, each community health worker is expected to oversee about four CHVs, who help with health promotion, prevention, monitoring, surveillance, response, and referrals [13]. The CHVs offer the first point of contact with the formal health system for many people and support the delivery of health services through community engagement, door-to-door visits, dispensaries, village health clinics (VHCs), and outreach clinics/posts [13]. However, the challenges of volunteer roles, social capital, and their connection to retention and attrition, as well as the identification of conflicting factors that motivate CHVs, persist [11, 12, 14].

The present study aimed to understand the motivations of individuals who became CHVs, those who remained, and those who dropped out (attrition) during a four-year MVIP programme in Malawi.

## Methodology

### Study setting

The study was conducted in nine districts (Ntchisi, Lilongwe, Mchinji, Balaka, Mangochi, Machinga, Phalombe, Chikwawa and Nsanje) in central and southern Malawi from November 2019 to March 2023 as part of the Malaria Vaccine Implementation Programme (MVIP) pilot project [15]. The MVIP evaluated the RTS, S/AS01 malaria vaccine using a cluster-randomized design, with 23 intervention clusters and 23 control clusters [15]. The MVIP was sponsored by the World Health Organization (WHO) and conducted in Malawi, Kenya, and Ghana to evaluate and assess the feasibility, safety, and effectiveness of delivering the RTS, S/AS01 malaria vaccine through the routine Expanded Program on Immunization (EPI) [15, 16]. The vaccine was introduced through the EPI program in twenty-three clusters, whilst the rest served as controls.

**Fig. 1:**
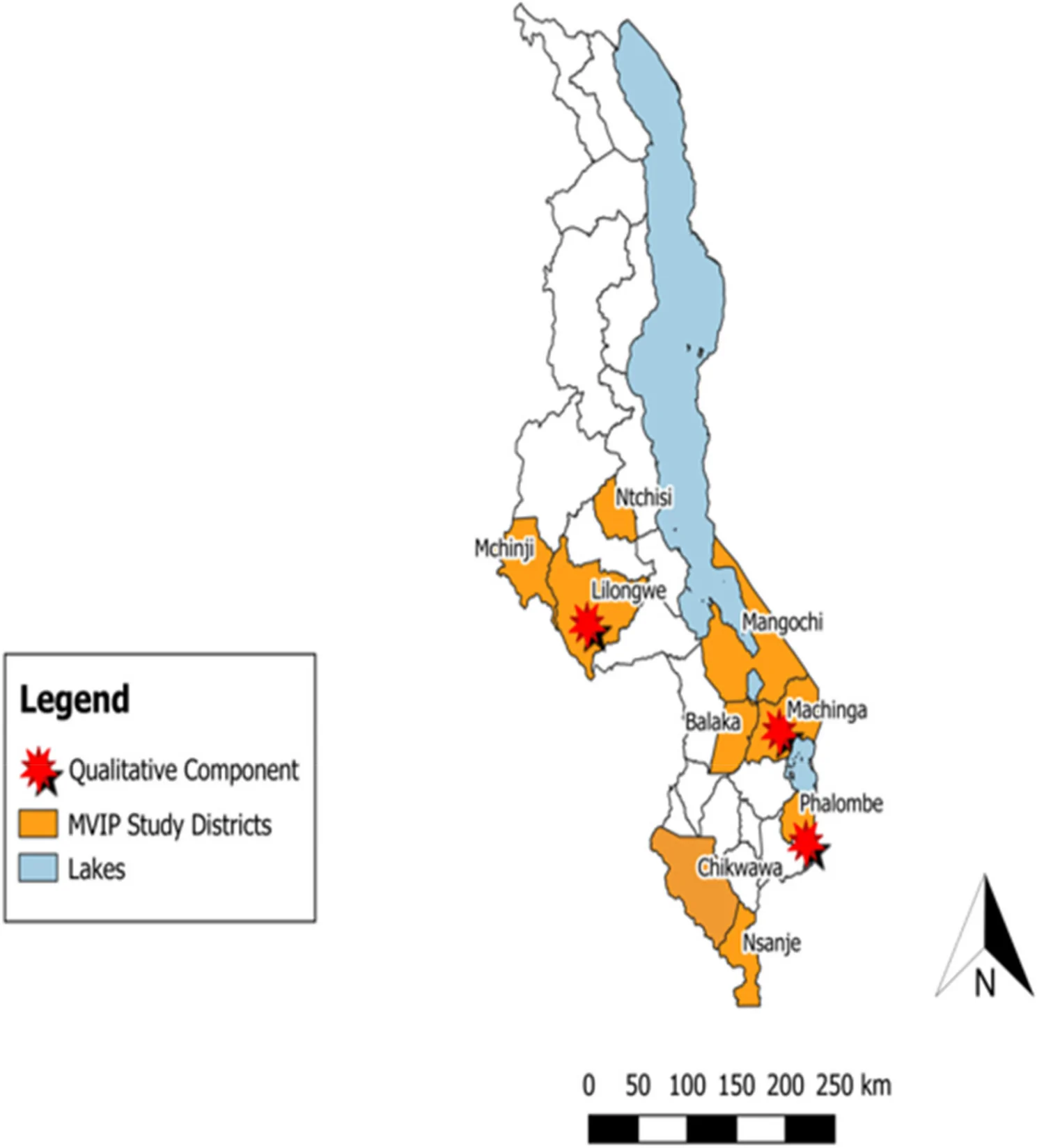
Is the map showing 9 MVIP implementing districts

In Malawi, nine moderate to high malaria transmission districts, with a total population of 6.2 million, were partitioned into 46 clusters [15, 17, 18] with a population of at least 130,000 people per cluster. See Fig. 1 for the map showing the 9 MVIP implementing districts. To conduct mortality surveillance, community health volunteers called CHVs were recruited to support local chiefs and community health workers in the mortality surveillance program. Selection of CHVs was based on one being a member of the village health committee, residing in the project area and being able to read and write. The CHVs attended a one-day training session on mortality surveillance. Their specific roles in the MVIP program included community sensitization, identification of new deaths, notifying the chiefs and community health workers of new deaths and supporting the community health workers with arranging verbal autopsies.

CHVs selected for the trial participated voluntarily through the MVIP program, and not all CHVs nationwide in Malawi. They received modest incentives designed to support their voluntary work and sustain their involvement in the field, rather than serve as formal labor compensation. These incentives included both monetary and non-monetary forms: a US $2 transport refund per verbal autopsy, US $5 for attending quarterly review meetings, US $0.7 monthly for airtime, a branded T-shirt and umbrella every two years, and certificates of training completion. Each quarter, the two best-performing CHVs in each cluster received an award comprising 2 kg packets of sugar and 30 tablets of bathing soap. The total cost of this award—defined as recognition or a prize given to acknowledge performance or achievement, often non-monetary or a one-off gift, sometimes with a small material value—was $6 per CHV.

### Study design

This study, nested within the MVIP, employed an inductively driven mixed-methods exploratory design using case studies as part of triangulation. The quantitative phase focused on socio-demographics, volunteer roles and experiences, motivation, and factors related to retention and attrition. The qualitative study included six cases of CHVs who dropped out and were reachable and willing to be interviewed to gather confidential, individual reasons for leaving. Additionally, focus groups were conducted with current program participants to gain a deeper understanding of group norms, shared practices, and factors influencing CHV motivation over time.

### Sampling and sample size

Purposive sampling was used to recruit participants for the qualitative component, which comprised five focus group discussions (FGDs) with CHVs (*n* = 50) for triangulation, and eight key informant interviews with health workers, opinion leaders, and programme staff for their perspectives on supervisory and programme processes. Six in-depth interviews (IDIs) were conducted with CHVs who had dropped out—many dropouts were spread out, hesitant to attend group meetings, or unreachable for focus group discussions (FGDs). These six IDIs are the only dropout participants who could be contacted and agreed to be interviewed, suggesting that discussing reasons for leaving in a group setting may suppress honest responses. Serving and dropout CHVs separately attended qualitative interviews. MVIP study coordinators and evaluation assistants assisted in locating the CHVs who had dropped, and the research team followed up with invitations to participate in the study. Thereafter, a cross-sectional survey involving 696 CHVs was conducted. The sample was drawn from a total of 2861 CHVs engaged to work in the 46 clusters of the MVIP programme. The range of CHVs per cluster was 31 to 187. The study employed the Probability Proportional to Size (PPS) sampling method to obtain a representative sample of CHVs within each cluster. Based on a 90% retention rate, a 95% confidence level, and 3% precision, the initial sample size was set at 384 participants. After accounting for clustering with a design effect of 1.8, the required sample increased to 691 participants. Consequently, the final sample of 696 community volunteers was sufficient for the study. MVIP study supervisors provided detailed lists of volunteers in their respective clusters, from which 696 CHVs were randomly selected. During data collection, when a selected CHV was unavailable, a replacement was randomly chosen from the same cluster.

### Data collection

Data for this study were collected periodically, including MVIP from 1 November 2019 to 31 March 2023. The qualitative research team comprised a graduate student completing a two-year master’s in public health and two senior qualitative researchers. The student participated in all research stages—interviewing, FGD facilitation, ethics, audio recording, transcription, translation, and coding—and conducted pilot interviews under the supervision of senior researchers during data collection and analysis. Qualitative participants, including CHVs, local chiefs, district health management committee members, and district supervisors for the MVIP program, took part in in-depth interviews and focus group discussions. The research team reached out to participants by phone to arrange interviews. These interviews were conducted over a month, alongside the quantitative data collection, which occurred two months after the four-year program data collection concluded.

Quantitative data was collected by 51 experienced evaluation assistants using a standardised tablet-based questionnaire, which took 30 to 45 min. The quantitative questionnaire data investigated if CHVs were involved in multiple volunteer projects, their reasons for participating in voluntary work, the incentives they would appreciate, the challenges and demotivating factors encountered during volunteering in the MVIP programme and strategies to be implemented to increase retention of volunteers. In IDIs with dropouts, no new reasons emerged after the sixth interview, indicating saturation.

The in-depth interviews and focus group guides were developed based on the quantitative questionnaire data. The questionnaire was pretested in two districts over three months as part of the MVIP pilot; the research team regularly revised it before expanding to nine districts. The qualitative tools were piloted in Blantyre and Lilongwe by a graduate student, reviewed with the supervisor and a senior social scientist for validity and question flow. The qualitative guides were derived from the quantitative instrument by converting key survey domains into themes and open‑ended questions, matching question order, and adding probes like “Can you describe that experience?” and “What did you mean by…?” to clarify responses and gather context. In-depth interviews lasted between 30 and 60 min, while group and key informant interviews ranged from 60 to 120 min (topic guide attached), exploring themes related to service to others, incentives, flexibility of working on multiple programmes, ridicule, and the absence of personal development and career growth.

Themes were identified both a priori—guided by key survey domains—and inductively, with refinement during initial analysis and subsequent feedback sessions to ensure that all discussions were considered [19]. FGDs with CHVs showed repetitive themes after the fourth, with the fifth confirming patterns and minor details. No new programme-level themes emerged after the seventh KII, and the eighth confirmed existing categories, indicating saturation across all methods [19].

Interviews were conducted with volunteers, district supervisors for the MVIP, and key informants from health facilities at central locations such as seminar rooms within health facilities, school blocks, or under-five shelters. Depending on participants’ preferences, interviews were held in either Chichewa or English. All interviews with village headmen took place in their homes in the local language, Chichewa.

### Data processing and analysis

For qualitative data analysis, digital data files containing in-depth key informants and group interviews were transcribed verbatim by three research assistants, and data were entered in Nvivo 11. Codes and themes were discussed in-depth between the authors and research assistants, compared with literature and theories in iterative ways to enhance interpretation. The first and second authors selected notable quotes and further discussed them with the rest of the team based on their relevance to the themes and translations in each paragraph. Key themes were matched with quantitative findings at the point of analysis and interpretation to highlight areas of convergence and divergence [20].

In quantitative data analysis, we reported baseline characteristics of the CHVs using proportions and percentages if categorical. Continuous data was summarized using mean and standard deviation if normal, medians with range if skewed. The primary outcome was retention -- defined as whether a CHV was willing to stay in the programme. “Still on the program” meant a CHV who, at late-phase assessment, (1) was on the district roster, (2) reported providing services in the past three months, or (3) was confirmed active by a supervisor. Originally, the study aimed to assess factors associated with retention among the CHVs using logistic regression models. However, nearly all CHVs indicated they were willing to stay on the program and therefore modelling was not feasible. These analyses used Stata SE version 15.1 (Stata Corp., College Station, TX) [21].

### Study conceptual framework

The study utilised the concept of social capital based on three constructs: roles, accompaniment, and empowerment. We used the social capital framework as an organising analytic lens throughout coding, analysis, and interpretation [19]. The framework guided initial code development, structured comparisons across cases, and anchored the interpretation of how relational resources influenced community health volunteer retention and attrition. This enabled a rigorous analysis of the various types and levels of resource support that affect CHV retention and attrition. The social capital framework refers to the resources derived from a network of relationships, care, concerns, compassion and trust that assist individuals in problem-solving and accomplishing tasks [22, 23]. Limited studies have examined social capital and its relationship to volunteer roles, empathy, responsibilities, and competing factors in explaining retention and attrition in Malawi [14, 23, 24].

## Results

### Description of participants

**Fig. 2:**
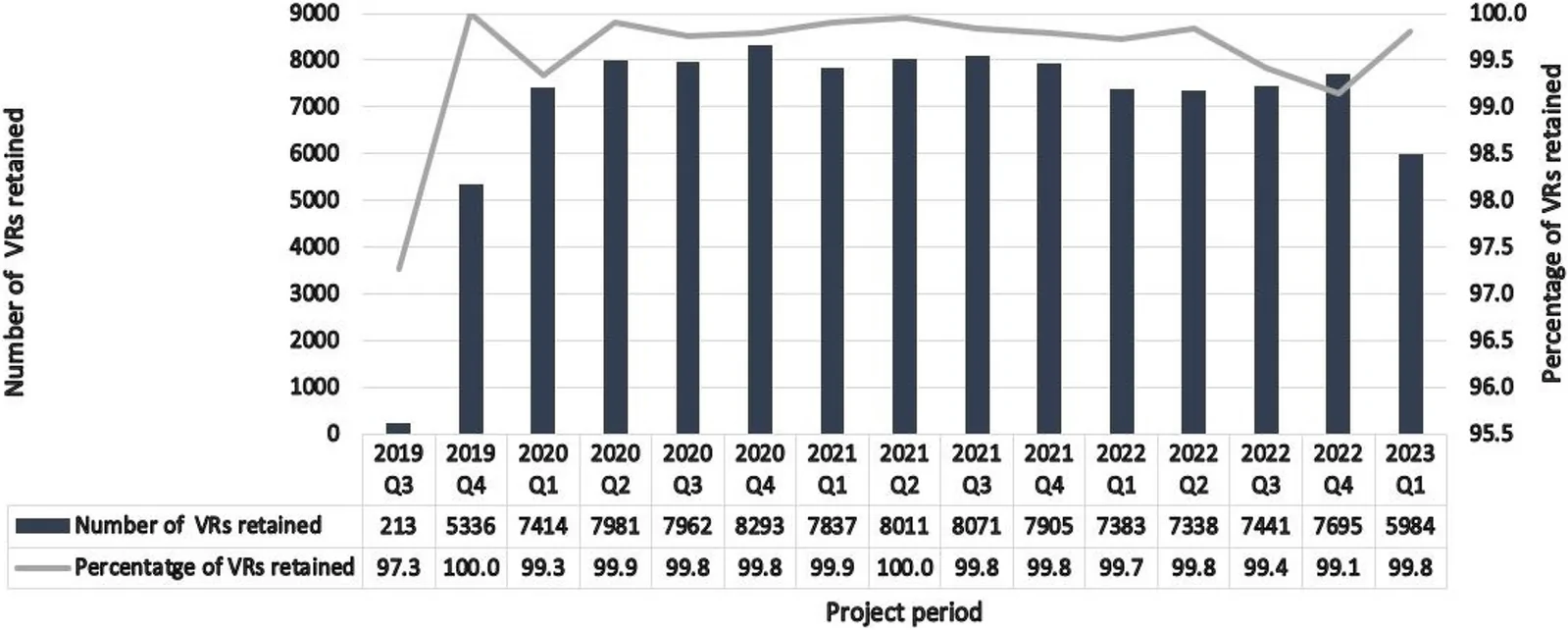
Showing number and percentage of CHVs retained throughout the MVIP program Nov 2019- March 2023

Overall, from programme inception, by March 2023, 89.7% of CHVs were still on the program, and 10.3% had dropped out. The number of CHVs generally remained constant throughout the study period. Figure 2 shows the retention of CHVs throughout the program from November 2019 to March 2023. The average age of CHVs who remained in the study was 41.9 (SD = 9.2) years, and the majority were male (58.2%), married (87.8%), and had either primary (52.7%) or secondary (46.7%) education. The CHVs had multiple sources of income: 92.0% were farmers, and 33.5% were vendors [involved in various businesses], among others. Table 1 below summarises the baseline and demographic characteristics of CHVs.

**Table 1:** Below summarises the baseline and demographic characteristics of participants

Variable	Category	*n* = 696, (%)
Age category, *n* (%)	18 to 24	5 (0.7)
	25 to 44	450 (64.7)
	> 45	204 (29.3)
Gender, *n* (%)	Female	291 (41.8)
Marital status, *n* (%)	Single	25 (3.6)
	Married	611 (87.8)
	Other	60 (8.6)
Education, *n* (%)	Primary	367 (52.7)
	Secondary	325 (46.7)
	Other	4 (0.4)
Religion, *n* (%)	Islam	147 (21.1)
	Christian	535 (76.9)
	Other	14 (2.0)
Other occupation, *n* (%)	Farmer	640 (92.0)
	Vendor	233 (33.5)
	Formal job	20 (2.9)
	Other	68 (0.6)
Duration of staying in the project area (years)	*≤* 5 years	26 (3.7)
	> 5 years	670 (96.3)
Hours spent on the project per week	Less than 5 h	357 (51.3)
	5 to 10 h	279 (40.1)
	11 to 15 h	39 (5.6)
	16 to 20 h	13 (1.9)
	More than 20 h	8 (1.2)
Prior experience in volunteering	Yes	635 (91.2)

### Retention of community health volunteers

The CHVs who completed the late-phase survey (695 out of 696), representing 99.8%, indicated their intention to stay and continue participating in the MVIP program. The majority of the CHVs (91.4%) indicated that they spent less than 10 h per week working on the program. CHVs who spent more than 10 h had more than the prescribed four villages per CHV or larger villages to service. When asked about their motivation to stay, or the factors without which they would not want to stay, CHVs mentioned incentives such as small monetary gains or material support—like T-shirts, plastic folders, and umbrellas—as reasons for volunteering in the MVIP program. (84.5%), transport and airtime allocation (76.4%), recognition e.g. acknowledging and celebrating CHVs hard-work and credibility through certificates or incentives (46.2%) and exchange visits e.g. organizing meetings in other districts for learning purposes (54.2%) as some of the motivating factors to remain as volunteers. CHVs from Chikwawa (59.2%) and Nsanje (47.2%) districts reported a high preference for salary as an incentive. Table 2 shows the facilitating factors for retention.

**Table 2:** Shows the motivation and facilitating factors for retention

Facilitating factors contributing to volunteer retention	Total *N*=696	Ntchisi	Lilongwe	Mchinji	Balaka	Mangochi	Machinga	Phalombe	Chikwawa	Nsanje
Provide incentives (T-shirts, airtime, umbrellas)	588(84.5)	51 (60.0)	124(82.7)	60(90.9)	50(89.3)	83 (93.3)	85 (87.6)	36 (70.6)	46 (93.9)	53(100)
Provision of timely transport refunds &airtime allocation	532(76.4)	37 (43.5)	108(72.0)	54(81.8)	52(92.9)	71 (79.8)	75 (77.3)	37 (72.6)	48 (98.0)	50(94.3)
Pay salary	247(35.5)	23 (27.1)	62 (41.3)	10(15.2)	19(33.9)	24 (27.0)	43 (44.3)	12 (23.5)	29 (59.2)	25(47.2)
Give certificates of recognition	249(46.2)	28 (32.9)	55 (36.7)	18(41.9)	14(63.6)	20 (71.4)	40 (69.0)	20 (39.2)	22 (44.9)	32(60.4)
Small manageable area (average of 4 villagesper CHV)	275(39.5)	20 (23.5)	53 (35.3)	31(47.0)	10(17.9)	52 (58.4)	61 (62.9)	19 (37.3)	16 (32.7)	13(24.5)
Exchange visits to other districts	377(54.2)	40(47.1)	58(38.7)	35(53.0)	33(58.9)	72 (80.9)	78 (80.4)	24 (47.1)	5 (10.2)	32(60.4)
Opportunities to rest and socialize with theircounterparts	154(22.1)	12(14.1)	36(24.0)	19(28.8	3(5.4)	25 (28.1)	28 (28.9)	18 (35.3)	10 (20.4)	3 (5.7)﻿
Other (e.g. Provision of allowances/refreshments at review meetings, including bicycles, phones, etc.)	124(17.8)	26(30.6)	33(22.0)	3 (4.6)	15(26.8)	3 (3.4)	3 (3.1)	13 (25.5)	6 (12.2)	22(41.5)

### Qualitative findings

Out of the 64 participants in the qualitative study, the CHVs had a median age of 39 years (range: 24–63). The key stakeholders had a median age of 44 years (range: 32–70). The CHVs had between 3 and 12 years of formal schooling: had a minimum education level of standard three in primary school (equivalent to completion of three years of primary school), and a maximum education level of form 4 in secondary school (equivalent to completion of four years of secondary school) and obtained a Malawi secondary school leaving certificate. Some key stakeholders had completed early primary school, while others held university or college degrees. The CHVs served for at least two years, with the longest-serving volunteers having served for four years under the MVIP and over twenty years in other government, private, and NGO programs. Most key stakeholders had held their posts as district coordinators for more than five years, while local leaders had served for more than ten years. Three main themes emerged as findings that influenced retention: service to others, incentives, and flexibility to work in multiple development programmes. The factors associated with attrition included the absence of personal and career development and ridicule from community members. Based on the analysis of social capital and data interpretation, we recognize that serving and accompanying other social and health actors, empowering CHVs through incentives and their involvement with other NGOs are crucial for CHVs to achieve their desired benefits and goals. However, there are several conflicting issues to consider: (1) incentives, (2) ridicule from community members, and (3) lack of individual development and career growth, which greatly contribute to the attrition of CHVs. Table 3 shows the main themes emerging from group and individual interviews.

**Table 3:** Key themes from the study

Main themes	Sub Theme	Description	Quotes
Retention	Service to others	Compassion to serve others	*I am proud of volunteering even if I am not paid for it. I am saying this because I can assist in improving our people’s well-being and health. FGD CHVs_LL*
	Incentives	Cash, duty facilitation allowances, materials, salaries and t-shirts.	*What empowers us is that when we have conducted the review meetings*,* they could provide us with allowances. The airtime and t-shirts that we received empowered us*. FGDs CHVs PD
	Flexibility of working on multiple programmes	Volunteer involvement with other NGOs	*So*,* I worked with the organization for many years. The chiefs knew*,* and whenever another organization came in*,* they just recommended us as volunteers for that other organization. FGD_CVHs_SE*
Attrition	Ridicule	Community members making fun of some aspect of another, involving sarcasm and degradation, rude comments influencing dropouts	*Some call us grave diggers*,* but we do not mind that. However*,* some people (CHVs) are susceptible to insults or being called grave diggers; they think of quitting*. IDI 02_CHV_LL
	Absence of personal development and career growth	Opportunities to develop new skills and work	*Being a volunteer is hard work. It needs dedication. We expect to get noticed by other organizations to improve ourselves. We do not expect payment but desire to develop our areas and make progress as a community and as individuals*. IDI_05 CHV_SE

### Motivators and demotivating factors of retention

#### Passion for serving others

Most volunteers stated that their main motivation was a willingness to sacrifice their time, with some foregoing income‑generating activities to volunteer. One explained, “I close my grocery shop to do volunteer work” (IDI‑02‑CHV‑MHG), demonstrating the trade‑offs volunteers make to remain active. Others explicitly linked this sacrifice to a desire to help the community, gain experience, and show concern, care, and compassion in serving and guiding their communities.

CHVs mentioned that they worked on several social health initiatives and programs to enhance skills as a long-term commitment to the community. Almost all CHVs believed they were trusted community resources and networks that accompanied social and health actors in providing health information and support.

*Village Health Committees chose CHVs based on criteria such as good reputation*,* dependability*,* trustworthiness*,* and access to phones for communication. Many of the volunteers selected were already engaged in community programs with other organizations* (KII, MoH Coordinator).

A *CHV performs various duties*,* including protecting and accompanying our co-workers --HSAs. Sometimes*,* HSAs face challenges and distrust while carrying out their duties. However*,* the presence of CHVs during these times ensures the safe delivery of health services in the community for the benefit of both the community and the HSAs (*FGD, CHVs, PH)

#### Incentives and duty facilitation allowances and materials

All the volunteers confirmed they were not paid a salary or monthly remuneration. They did not consider the money or incentives given as compensation for their responsibilities. Instead, the materials and supplies provided to the CHVs as duty facilitation allowances were referred to as “incentives”. Therefore, the timely provision of material benefits such as food, transport reimbursement, airtime, T-shirts, umbrellas, and folders was mentioned as a motivating factor for remaining a volunteer. These incentives fostered volunteerism and empowered CHVs by providing support from external resources to help improve their personal and working conditions and fulfil their community functions.

*What empowered us was that they could provide us with airtime and t-shirts when we conducted the review meetings. Considering that we still need to travel during the rainy season to do our work*,* the umbrellas they gave us will protect us from the rain.*

*The T-shirts empowered us because the homeowners had no concerns or questions. It was clear we were CHVs. The T-shirt instantly identifies us as being there for our health-related duties* *(FGD*,* CHVs*,* LL).*

Most long-serving volunteers realized that their roles and opportunities only sometimes result in tangible financial gain. When asked how much would be a good incentive, CHVs indicated that they could not control volunteer allowances or monetary incentives, as these are considered administrative matters for the program.

For some, volunteerism was a legitimate means of survival, a livelihood strategy, and they expected reasonable financial incentives. Even though they realized they could not dictate allowance or salary amounts, they expected monetary incentives and/or salary to align with the current economic environment. They mentioned a monthly allowance or salary as their most significant motivator to meet their personal and family needs and well-being.

*As it stands*,* prices keep on increasing. It is important to consider the devaluation. Moreover*,* if we give someone $23 per month*,* how would that support an unemployed? The amount is inadequate. Since the person is a volunteer*,* they only receive whatever you provide. Volunteers cannot ask for more or make demands* (IDI, CHV1, SW)

This view suggests that financial facilitation must be both predictable and sufficient to offset opportunity costs if volunteerism is to remain a viable survival strategy.

#### Flexibility of working on multiple programmes

CHVs of all ages shared strategies for effectively managing their time while fulfilling their responsibilities to the community and their families. Some volunteers mentioned that they had dedicated themselves to more than two NGOs, allowing them to become full-time volunteers. This ability to volunteer for multiple organizations was a valuable asset, as it enabled them to tap into ‘existing and hidden’ social and health networks and adapt to local conditions, ensuring their own survival, retention, and well-being.

*I started as a volunteer extension worker and then joined the health centre in my village. They noticed my potential and assigned me different tasks*,* which led me to become a fully-fledged volunteer* (FDG_CHVs_LL).

Many older volunteers mentioned splitting their time between various activities, such as working with multiple NGOs, farming, managing village banks, and fulfilling household duties. Some had also worked as extension workers on farming and water, sanitation, and hygiene (WASH) programs. In contrast, others primarily supported health and rights-based NGOs XX and HIV and nutrition care groups, acting as experts, clients, and counsellors, among other roles. This increased their social participation, enhanced their confidence, and fostered a sense of purpose and connection to the community.

*We have allocated our time to different activities. In the morning*,* we leave early to go to the farm. We plan to work on the MVIP project activities*,* meet the chief*,* and review the registers and reports. Afterwards*,* you can also visit those in care groups [other NGO programmes]. We do not have time to sit idle; we will do our farming activities later before the sun sets* (FGD_CHVs_SE).

### Factors influencing attrition of volunteers

#### Ridicule – use of derogatory names by the community

The CHVs were popularly referred to as *‘Alangizi a imfa’* (counsellors of death) because of their role in reporting new deaths in the community. This displeased some CHVs and led them to resign from their positions. Those who had been volunteers for a shorter period (less than 3 years) felt belittled by such names. However, experienced volunteers welcomed these names as a badge of honour and an endorsement of their community responsibilities.

*Despite the challenges*,* we*,* the CHVs*,* were dedicated to our role. We were given the nickname anthu oyendetsa maliro [CHVs as coordinators of death processions] because we documented a thorough history of each deceased person on paper. One report was sent to the chief*,* while the other was given to the gravediggers. Additionally*,* we diligently recorded every funeral in the communities* (IDI, 02_CHV_SE).

*Talking about death can be difficult. Some community members negatively perceive CHVs*,* believing their work solely focuses on death. As a result*,* they are often ridiculed and referred to as “Alangizi an imfa” or "* counsellors of death *“. When they see us*,* they assume there has been a death in the community. This association with death is something we*,* as CHVs*,* constantly face.* (FGD_01_CHVs, SW)

#### Lack of personal development and career growth

One reason for dropping out was that volunteers perceived the work as unpromising and without the potential for personal and career development. A few CHVs left their posts to pursue better opportunities.

*However*,* it is rare for CHVs to drop out without a reason. Most of them said they were moving out*,* getting married*,* or leaving for business or work. But*,* it is not a common occurrence for CHVs to drop out* (KII-01-EA-PE)

*Some quit working as CHVs because they found a job at the hospital*,* but some stop working as CHVs because their husbands do not see any benefits* (IDI, 02 CHV, LL).

## Discussion

This research was built upon the social capital approach introduced by Mohajer and Singh (2018) and explored the factors influencing motivation, attrition, and retention of volunteers [23]. The study suggested that volunteers who viewed their work as a calling were more likely to find satisfaction in their experiences. As a result, they were more likely to remain in their roles, even if they were not adequately compensated or experienced ridicule. The research identified various factors, needs, and expectations related to the retention and attirition of CHVs and the community programmes and highlighted the significance of serving, accompanying, and empowering others. Although some volunteers may not have met all their needs and expectations, they still chose to stay. Only a few left their positions for other opportunities due to their unique motivations centred around service, accompaniment, and empowerment.

This study shows that almost 90% of the community health volunteers continued to offer services for more than four years in the program. This level of retention is consistent with few studies on the retention of CHVs in sub-Saharan Africa, where on a volunteer-government health promotion program, about 90% of volunteers were retained between 2 and 5 years [6, 8, 25, 26]. The high retention rate can be attributed to the following: (1) CHVs who were experienced and were volunteering on other programmes, meaning that they were already exceptional members of the community with support from the community leadership; (2) living and serving within the vicinity and accompanying local CHW/HSAs (3) provision of relevant incentives/ duty facilitation materials. High CHV retention (~ 90% over 4 years) indicates Malawi can expand volunteer services if policies provide predictable incentives, supervision, training, and integration into health systems support.

Most volunteers attributed their continued involvement to personal and professional factors and to altruistic values —such as serving others and giving back to communities. The CHVs’ compassion drove their willingness to alleviate suffering and improve health outcomes, particularly with limited resources and without pay, a pattern consistent with other reports from Africa [27–30]. The literature portrays CHVs as usually compassionate and highly dedicated to the communities and volunteer work, which provides them with satisfaction and value [29, 31, 32]. CHVs attribute altruism, community standing, and professional growth as reasons for long service. Policy should protect and boost these non-financial motivators while providing support like facilitation, supervision, training, and pathways to paid roles to sustain and scale volunteer programs.

Additionally, the CHVs in our study demonstrated a willingness to switch between NGOs to do what is right and find purpose and meaning in their lives. This study recognized that CHVs are skilled individuals who, accompanied CHWs/HSAs in the community and worked effectively with diverse groups and settings. This ability allowed them to maintain continuity in their volunteer work across different organizations. However, questions frequently arise regarding their sense of responsibility, the impact on their time and family obligations, program outcomes, and the challenges associated with increased burnout [33, 34]. Burnout can occur due to the absence of incentives, capacity building, and ridicule, as these dissatisfactions outweigh the benefits of the position. Fartuk & Barnard (2021) worry about adding new tasks to the responsibilities of CHVs, which may change the costs and benefits of volunteering [29]. Although there is a misconception that CHVs can easily stop their activities, they invested a lot of time, effort, and commitment and may continue to volunteer despite burnout. It is inaccurate to assume that volunteer work is purely voluntary, so burnout among volunteers deserves more attention.

Volunteerism is not always done without expecting something in return. In this study, all CHVs received duty facilitation allowances and materials, which they identified as ‘incentives’. This shows that CHVs received benefits that helped them fulfil their roles and reduce attrition, but they are not a perfect solution [35, 36]. Many organizations do not provide allowances due to concerns about funding sustainability. This finding is consistent with previous studies that have reported attrition rates ranging from 3% to 77% [6, 25, 26, 37]. The provision of duty facilitation allowances to CHVs remains controversial for several reasons: (1) they may not be sufficient, (2) only a limited number of people may receive the allowances, (3) the allowances may be irregular, or (4) the allowances may eventually stop [27, 38–40]. Additionally, CHVs in the MVIP program from two districts, Chikwawa and Nsanje, requested that they be given salaries as a rightful incentive to work on the program and to encourage volunteer retention. Therefore, organizations that engage CHVs are encouraged to work with community leaders to select volunteers and provide non-monetary and monetary incentives to reduce volunteer attrition.

Regarding ‘flexibility volunteering’, the volunteers in the current study took on various roles, such as conducting weekly and monthly visits, keeping records, and caring for their assigned households within their catchment areas. This demonstrates that volunteerism is manageable, and attrition was lower when volunteers were flexible in engaging with other NGOs. Consequently, they allocated time to run their small businesses, engage in farming, and attend village banks to support their livelihoods. In the context of Malawi, flexible volunteering allowed CHVs to select the tasks they wanted to undertake and determine the frequency of their involvement, fostering empowerment and task shifting. A systematic review also recognized the diverse roles and contributions of CHVs in different settings, which enhanced retention and development beyond health [6, 28, 39]. Despite CHVs being helpful across multiple organizations, their efforts must be combined with those of community leaders and other partners to ensure long-term success, improved outcomes, and the addressing of attrition.

This study also showed that volunteers often face name-calling and ridicule from their communities, which creates stigma around their work. This stigma made it difficult for younger volunteers – those who had worked for less than three years- to continue their work, ultimately forcing others to drop out. Additionally, to the older CHVs (who worked four years and above), the combination of the absence of career development, ridicule and stigma from the communities were mentioned only occasionally or seldom as demotivating CHVs to drop out of volunteer programs [4, 12, 28]. Likewise, fewer previous studies on volunteers frequently cited stigma and harassment from communities as obstacles limiting their flexibility to pursue other endeavours, leading to increased attrition [4, 8, 37]). Although community ridicule may not be the main reason for the laughter surrounding volunteers, it still contributed to stigmatized identities, which in turn had consequences for volunteer recruitment, satisfaction, and retention. Nichols (2024) argues that it is disempowering when workers feel undervalued [34]. Other challenges related to attrition factors (misplaced emphasis on salaries, long distances between participants, lack of trainings, workshops and timely support) are similar to those identified in various studies [28, 41, 42]. These challenges affect all volunteers living in impoverished settings in general and need to be addressed in order to improve their retention.

This study showed that the lack of personal and career growth opportunities contributed to volunteer attrition. CHVs anticipated that their volunteer work would enhance their prospects for education, upgrading and better employment opportunities within or outside the implementing organisations. The absence of personal and career growth has also been widely discussed as a leading cause of high attrition rates among trained CHVs [30, 35]. This, in turn, has a negative impact on motivation and job satisfaction. To address this issue, especially in resource-limited settings, it is crucial for implementing organizations and donors to be transparent and clearly communicate the educational and career growth opportunities available to CHVs. Emphasizing study objectives and outcomes is also important. Understanding the motivations of volunteers is important for social, health, and research organizations to develop effective strategies for volunteer recruitment and retention.

### Study strengths and limitations

This study had several key strengths—first, using a mixed methods design allowed for robust results. Additionally, the study provided a deeper understanding of the quantitative findings by explaining volunteer retention and attrition using the social capital framework in a low-resource setting. The researchers also worked with existing community-based structures, such as local leadership and the community village, to identify experienced volunteers who remained engaged throughout the implementation period. However, one limitation of the study was that it did not quantitatively capture individual-level reasons for attrition during the implementation period, which affected the robustness of the triangulation and interpretation of the findings.

## Conclusion

The CHVs demonstrated a strong commitment to serving and supporting others despite coming from marginalized communities and facing challenges in maintaining their livelihoods. Those who viewed their work as a service were more likely to find satisfaction and remain in their roles, even in the absence of adequate compensation and despite community ridicule and stigmatization. The study identified facilitating factors and barriers that influenced the retention and attrition of CHVs and community programs, highlighting the importance of duty facilitation allowances and materials, flexible volunteering, community ridicule, and lack of career growth. While some volunteers did not have all their needs and expectations met, they chose to continue their service. Only a few CHVs relinquished their positions to seek educational and better job opportunities driven by personal aspirations.

## Supplementary Information

Supplementary Material 1.

## Data Availability

The authors will make the data supporting this article’s conclusions available without undue reservation.

## Abbreviations

CHV: Community Health Volunteer
CHW: Community Health Worker
CHV: Community Health Volunteers
DHMT: District Health Management Team
EAs: Evaluation Assistants
FGD: Focus Group Discussion
HSA: Health Surveillance Assistant
IDI: In-depth Interviews
MVIP: Malaria Vaccine Implementation Programme
NGO: Non-governmental Organisation
VHC: Village Health Committees
VRs: Village Reporters
