# Supplementary material for: Retention and attrition of community health volunteers in rural Malawi; a mixed-methods study
Source: BMC Public Health. 2026 Jul 1;26:2644. doi: 10.1186/s12889-026-28321-0 (PMC13595695; doi:10.1186/s12889-026-28321-0)
Supplement: Supplementary file 1 [file 12889_2026_28321_MOESM1_ESM.docx]

**Supplementary File: Topic guide**

**FOCUS GROUP DISCUSSION AND KEY INFORMANT GUIDE**

**Volunteers/Community FGD Guide**

|  | **FGD #: District:**  **Date: Start time: End time:**  **AM/PM**  **AM/PM** | | | | | |
| --- | --- | --- | --- | --- | --- | --- |
|  | | **Age** | **Cadre** | **Education** | **Years in voluntary role/work** | **(Women only group, men-only group, mixed-gender group)** |
| 1 | |  |  |  |  |  |
| 2 | |  |  |  |  |  |
| 3 | |  |  |  |  |  |
| 4 | |  |  |  |  |  |
| 5 | |  |  |  |  |  |
| 6 | |  |  |  |  |  |
| 7 | |  |  |  |  |  |
| 8 | |  |  |  |  |  |
|  | **Education:** 1= None, 2=Junior primary, 3= Senior primary, 4=Junior secondary, 5=Senior secondary, 6=University degree, 7=Other certificates and diplomas, (specify), 8=Other (specify) | | | | | |
|  | **Instructions for FGD Moderator:**   - This guide should help you to initiate and moderate the discussion, which should flow like a normal discussion - rather than a question-answer session - The guide is designed for a group of 6-12 community members to be conducted at a school or community. - Participants must be aware that focus group discussions will take between 30- 60 minutes - Notes should be taken throughout the sessions. - Ensure privacy (including audio privacy) for the discussion. - Establish some ground rules about the confidentiality of what is discussed within the group. | | | | | |

**Guiding Questions**

**Perceptions of Volunteerism**

1. How many of us have volunteered before?
2. In your view, what does volunteering mean?
3. In your own words, describe what the MVIP project is about.
4. You chose to volunteer for the MVIP project. Can you share your reasons for this decision?
5. In your own words, please describe your activities on the MVIP project.
6. What were your specific roles on the project?

**Motivation for volunteer work**

1. What were the benefits of becoming a volunteer under the MVIP project?

2. What challenges did you encounter during the MVIP project?

3. How were you compensated for your work? Please elaborate on the incentives provided.

4. What incentives would you have preferred to receive?

**Volunteer retention/turnover**

1. 1. Does anyone know someone who was once a volunteer on the project but has since left? Probe the reasons for their departure.
2. For those who have previously volunteered on this project:
   a. What motivated you to become a volunteer for that project?
   b. Can you describe your role in social and health-related projects?
3. What were the key challenges you encountered during that project?
4. What do you think should be done to attract new volunteers and retain existing ones?

**Volunteering and remuneration**

1. How were you compensated for your time in the project?
   a. Probe on monetary compensation, service support, materials, or other forms of remuneration you received.
   b. Probe on the incentives you receive as a volunteer in that project?
   c. What types of incentives would you have preferred to receive?
2. In this community, what incentives are typically offered to individuals who volunteer for projects?
3. In your own words, what are the challenges associated with being a volunteer?
4. How can we encourage people to continue volunteering?
5. Do you have any recommendations for volunteer work? Probe.
